# Prevention of mitochondrial genomic instability in yeast by the mitochondrial recombinase Mhr1

**DOI:** 10.1038/s41598-019-41699-9

**Published:** 2019-04-01

**Authors:** Feng Ling, Elliot Bradshaw, Minoru Yoshida

**Affiliations:** 1 Chemical Genomics Research Group, RIKEN Center for Sustainable Resource Science, Hirosawa 2-1, Wako, Saitama 351-0198 Japan; 2 Graduate School of Science and Engineering, Saitama University, Saitama, 338-8570 Japan; 3 Department of Biotechnology, Graduate School of Agricultural Life Sciences, the University of Tokyo, Tokyo, 113-8657 Japan; 4 Collaborative Research Institute for Innovative Microbiology, the University of Tokyo, Tokyo, 113-8657 Japan

## Abstract

Mitochondrial (mt) DNA encodes factors essential for cellular respiration, therefore its level and integrity are crucial. *ABF*2 encodes a mitochondrial DNA-binding protein and its null mutation (*Δabf2*) induces mtDNA instability in *Saccharomyces cerevisiae*. Mhr1 is a mitochondrial recombinase that mediates the predominant form of mtDNA replication and acts in mtDNA segregation and the repair of mtDNA double-stranded breaks (DSBs). However, the involvement of Mhr1 in prevention of mtDNA deletion mutagenesis is unknown. In this study we used *Δabf2 mhr1-1* double-mutant cells, which lose mitochondrial function in media containing fermentable carbon sources, to investigate whether Mhr1 is a suppressor of mtDNA deletion mutagenesis. We used a suppresivity assay and Southern blot analysis to reveal that the *Δabf2* mutation causes mtDNA deletions rather than an mtDNA-lacking (ρ^0^) phenotype, and observed that mtDNA deletions are exacerbated by an additional *mhr1-1* mutation. Loss of respiratory function due to mtDNA fragmentation occurred in ∆*mhr1* and ∆*abf2 mhr1-1* cells. However, exogenous introduction of Mhr1 into *Δabf2 mhr1-1* cells significantly rescued respiratory growth, suggesting that Mhr1-driven homologous mtDNA recombination prevents mtDNA instability.

## Introduction

The mitochondrial genome (mtDNA) encodes rRNAs, tRNAs and electron transport chain subunits essential for cellular respiration. Thus, maintenance of mtDNA level and integrity are crucial for healthy respiratory function^1–4^. In human cells, high mtDNA deletion levels can cause mitochondrial dysfunction and have been linked to neuromuscular disorders^5,6^.

MtDNA is packaged into a nucleoprotein complex termed the mitochondrial nucleoid, which is regarded as the unit of mtDNA inheritance^7^. Abf2 is a key component of the nucleoid with a histone-like role^8,9^, and contains two high mobility group (HMG) domains for the binding and efficient packaging of linear double-stranded DNA without supercoiling^10^. Abf2 wraps and bends mtDNA, but is not required for the activity of promoters at *ori* sequences and has no transcriptional role in yeast^11–13^. Mutants lacking *ABF2* (*∆abf2*) display a loss-of-mtDNA phenotype^8,14–16^ when utilizing fermentable carbon sources for growth, but are able to maintain wild-type mtDNA in non-fermentable media, indicating that the requirement for Abf2 in ρ^+^ cells is conditional. The *∆abf2* phenotype is considered typical of nuclear gene mutations that affect mtDNA maintenance, since more than 100 nuclear genes that influence mtDNA integrity in yeast have been identified^1,2^.

*Mhr1-1* is a temperature-sensitive point mutation in the nuclear gene *MHR1*, which causes deficiency in mtDNA homologous recombination^17^. Mhr1 is a mitochondrial recombinase^18,19^ that acts in double-stranded break (DSB) repair, mediates the predominant form of mtDNA replication in ρ^+^ cells^15,17,20–23^ and increases mtDNA content without additional Abf2^21^. DSBs can be created at replication origin (*ori*) sequences by excision repair enzymes such as Ntg1^21,24^. Following procession of DSBs by the 5′-3′exonuclease activity of Din7^22^, 3′-single stranded DNA can be used by Mhr1 to form a heteroduplex joint, in which the 3′-single stranded DNA tail serves as a primer to initiate rolling circle DNA replication, which produces linear multiple-unit-sized mtDNA molecules, termed concatemers, that promote segregation of heteroplasmy towards homoplasmy^20^.

In this study, we investigate whether Mhr1-driven mtDNA replication and homologous recombination contributes to the maintenance of mtDNA content and genomic integrity in *mhr1-1* cells with a null *abf2* genetic background, which show a loss-of-mtDNA phenotype in fermentable media due to deletion mutagenesis.

## Results

### Double-mutant ∆*abf2 mhr1-1* cells rapidly lose respiratory function in fermentable media

**Figure 1:**
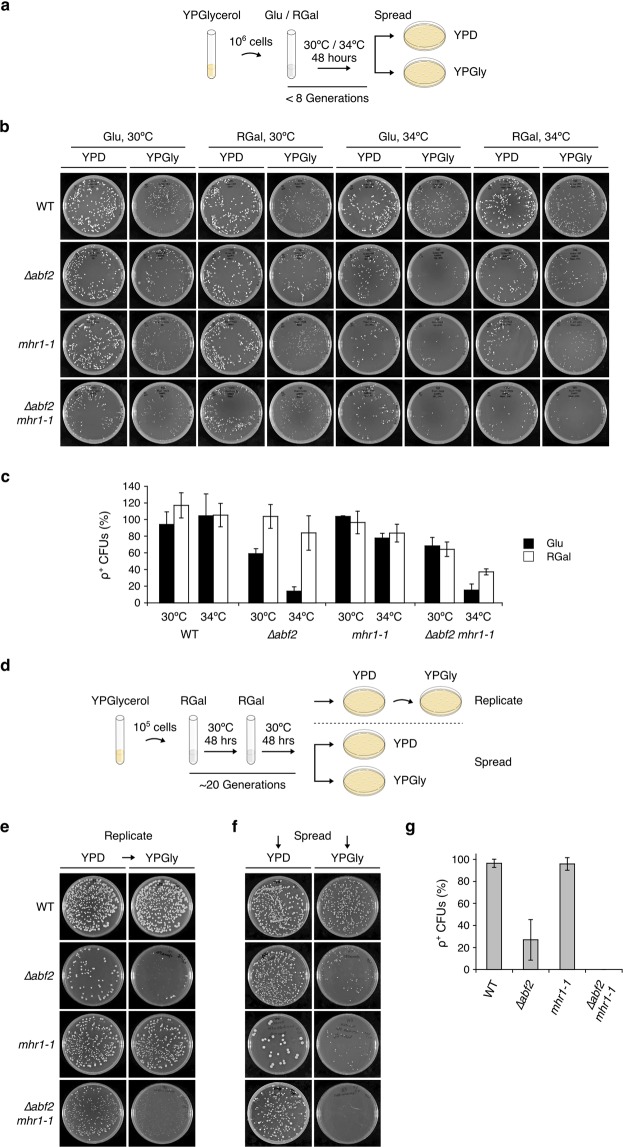
Loss of respiratory function in *∆abf2*, *mhr1-1* and *∆abf2 mhr1-1* cells. (**a**) Scheme of respiratory function assay. Cells were selectively pre-cultured in YPGly medium, then 10^6^ cells were transferred to Glu or RGal media and cultivated for <8 generations at 30 °C or 34 °C. Equal volumes of dilute culture were then spread onto YPD and YPGly plates to measure the proportion of CFUs retaining respiratory function. (**b**) Representative plate images of wild-type, *∆abf2*, *mhr1-1*, and *∆abf2 mhr1-1* CFU formation on YPD and YPGly plates following cultivation in Glu or RGal media at 30 °C or 34 °C. (**c**) ρ^+^ CFU formation rate based on *n* = 3 independent experiments described in (**a**). (**d**) Scheme of extended respiratory function assay. Cells were selectively grown in YPGly media, then 10^5^ cells were transferred to RGal media and cultivated for two consecutive 48-hour rounds (approximately 20 generations) at 30 °C. Equal volumes of dilute culture were then: (**e**) Spread onto YPD and YPGly plates, or (**f**) spread onto YPD plates, grown for four days, and then replica-plated onto YPGly plates. (**g**) ρ^+^ CFU formation rate from cells simultaneously spread onto YPD and YPGly, based on *n* = 3 independent experiments. All error bars represent ± SD.

In order to examine severely compromised mtDNA maintenance, we used *∆abf2* cells (Table 1), which display a well-documented loss-of-mtDNA phenotype upon cultivation in fermentable media^8,14,15^. In order to compare the extent of respiratory function loss in these backgrounds, we first selectively pre-cultivated wild-type (WT), single-mutant *∆abf2*, *mhr1-1*, or double-mutant *∆abf2 mhr1-1* cells in glycerol medium, a carbon source requiring mitochondrial respiration for its utilization. We then transferred the cells to synthetic complete fermentable media containing glucose (Glu) or raffinose and galactose (RGal) as carbon sources. While both Glu and RGal media are fermentable, the use of RGal allows for distinction from the transcriptional effects of glucose, which changes the global gene expression pattern^25^. We cultivated cells for nearly eight generations at 30 °C or 34 °C (Fig. 1a) and then spread equal amounts of dilute culture of each strain onto rich glucose (YPD) and rich glycerol (YPGly) plates (Fig. 1b). The proportion of colony-forming units (CFUs) that retained mitochondrial respiratory activity and were thus able to grow on YPGly, compared to the total number of CFUs on YPD, was quantified (Fig. 1c).

**Table 1:** Yeast strains used in this study.

Abbreviation	Strain	Nuclear genotype	Mitochondrial genotype	Source
WT/WT	CG380	*MAT*a/*MATα ade5*/*ade5* +/*his7 leu2*/*leu2 ura3*/*ura3 trp1*/*trp1*	[ρ^+^]	Stock culture
WT/*∆mhr1*	CG380 *∆mhr1*	*MAT*a/*MATα ade5*/*ade5* +/*his7 leu2*/*leu2 ura3*/*ura3 trp1*/*trp1 mhr1::LEU2*	[ρ^+^]	Stock culture
*mhr1-1*	FL67-1423 *∆ura3*	*MATα his1 trp1 ura3 can1 mhr1-1*	[ρ^+^ ω^+^ *∆ens*2 Oli_2_^R^]	^17^
*∆abf2 mhr1-1*	FL67-1423 *∆ura3 ∆abf2*	*MATα his1 trp1 ura3 can1 mhr1-1 abf2::KAN*	[ρ^+^ ω^+^ *∆ens*2 Oli_2_^R^]	This study
*∆abf2 mhr1-1/* pVT	FL67-1423 *∆ura3 ∆abf2*/pVT100U	*MATα his1 trp1 ura3 can1 mhr1-1 abf2::KAN* pVT100U (*URA3*)	[ρ^+^ ω^+^ *∆ens*2 Oli_2_^R^]	This study
*∆abf2 mhr1-1/* pVT-*MHR1*	FL67-1423 *∆ura3 ∆abf2*/pVT100U-*MHR1*	*MATα his1 trp1 ura3 can1 mhr1-1 abf2::KAN* pVT100U (*MHR1*, *URA3*)	[ρ^+^ ω^+^ *∆ens*2 Oli_2_^R^]	This study
	IL166-187	*MATα his1 trp1 can1*	[ρ^+^ ω^+^ *ens*2 Chl_321_^R^]	Stock culture
	IL166-187 *∆abf2*	*MATα his1 trp1 can1 abf2::KAN*	[ρ^+^ ω^+^ *ens*2 Chl_321_^R^]	This study
WT	IL166-187 *∆ura3*	*MATα his1 trp1 ura3 can1*	[ρ^+^ ω^+^ *ens*2 Chl_321_^R^]	This study
*∆abf2*	IL166-187 *∆ura3 ∆abf2*	*MATα his1 trp1 ura3 can1 abf2::KAN*	[ρ^+^ ω^+^ *ens*2 Chl_321_^R^]	This study
*∆abf2*/pVT	IL166-187 *∆ura3 ∆abf2*/pVT100U	*MATα his1 trp1 ura3 can1 abf2::KAN* pVT100U (*URA3*)	[ρ^+^ ω^+^ *ens*2 Chl_321_^R^]	This study
*∆abf2*/pVT-*MHR1*	IL166-187 *∆ura3 ∆abf2*/pVT100U-*MHR1*	*MATα his1 trp1 ura3 can1 abf2::KAN* pVT100U (*MHR1*, *URA3*)	[ρ^+^ ω^+^ *ens*2 Chl_321_^R^]	This study
	OP11C-55R5	*MAT*a *leu2 ura3 trp1*	[ρ^+^ ω^−^ *ens*2 Oli_1_^R^]	^17^
	YKN1423	*MATα leu2 ura3 met3*	[ρ^+^ ω^+^ *∆ens*2 Oli_2_^R^]	^18^
	YKN1423A-2	*MATα leu2 ura3 met3*	Normal suppressive [ρ^−^]	^47^
	YKN1423C-1	*MATα leu2 ura3 met3*	[HS ρ^−^]	^24^
	YKN1423 ρ^0^	*MATα leu2 ura3 met3*	[ρ^0^]	EtBr treatment of YKN1423

We observed that WT cells retained almost all respiratory function under each condition, while *∆abf2* cells showed remarkable loss of respiratory activity in Glu media, to 59.1 ± 5.9% ρ^+^ at 30 °C and 14.0 ± 5.3% ρ^+^ at 34 °C. On the other hand, cultivation of *∆abf2* cells in RGal medium resulted in a large proportion of cells retaining respiratory activity, forming ρ^+^ CFUs at rates of 103.9 ± 14.3% at 30 °C and 83.9 ± 20.7% at 34 °C. These observations appear consistent with the previously reported mtDNA instability phenotype of *∆abf2* cells in glucose^14^. In the *mhr1-1* background, a large proportion of CFUs retained respiratory activity, with ρ^+^ CFU formation rates of 103.9 ± 0.7% and 77.6 ± 6.0% ρ^+^ in Glu, and 96.5 ± 13.5% and 83.7 ± 10.6% ρ^+^ in RGal, at 30 °C and 34 °C, respectively. The slight decreases in respiratory activity of *mhr1-1* cells grown at 34 °C was previously observed as *mhr1-1* temperature sensitivity^17,20^. Furthermore, *∆abf2 mhr1-1* double-mutant cells displayed ρ^+^ CFU formation rates of 68.3 ± 10.4% and 15.4 ± 7.3% ρ^+^ in Glu, and 64.3 ± 8.8% and 37.2 ± 3.7% ρ^+^ in RGal, at 30 °C and 34 °C, respectively. Severe, temperature-dependent loss of respiratory function occurred in the *∆abf2* single-mutant in Glu but not RGal, while *∆abf2 mhr1-1* double mutant cells displayed an additive increase in temperature sensitivity in RGal. These results indicate that loss of mitochondrial function in this background occurs independently of glucose repression.

We next explored the effect of extended cultivation time on loss of cellular respiratory function. We pre-cultivated the four strains in YPGly medium, then transferred the cells to RGal medium and cultivated for approximately 20 generations (Fig. 1d). Finally, we spread the cells on YPD and subsequently replicated the colonies onto YPGly plates (Fig. 1e), or simultaneously spread equal amounts of dilute culture onto YPD and YPGly plates (Fig. 1f). In contrast to WT and *mhr1-1* single-mutant cells, which remained almost entirely ρ^+^, only 27.0 ± 18.4% of the *∆abf2* cells were able to form ρ^+^ colonies on glycerol plates following simultaneous spreading. Remarkably, none of the *∆abf2 mhr1-1* double-mutant cells were able to grow on YPGly plates after growth for approximately 20 generations in RGal (Fig. 1g). Therefore, extended cultivation of the *∆abf2* single-mutant in fermentable RGal media increases loss of cellular respiratory function, while the additional loss of Mhr1 function causes rapid and complete loss of cellular respiratory function^1,14^.

### Nucleoid numbers are significantly reduced in ∆*abf2 mhr1-1* cells

**Figure 2:**
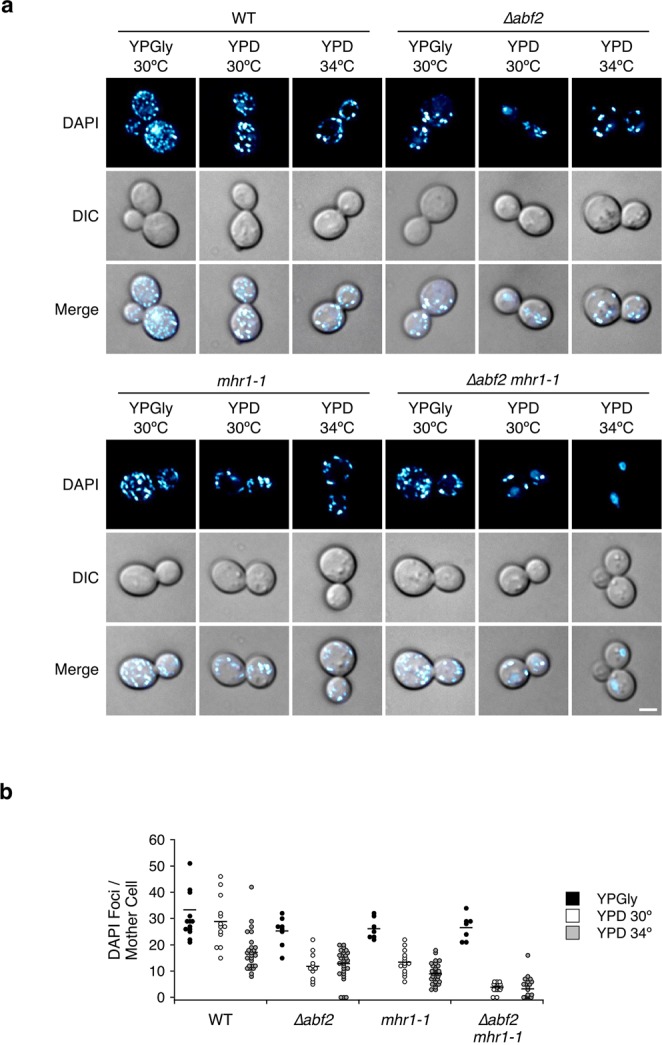
Reduction in mtDNA-derived DAPI signals in *∆abf2*, *mhr1-1* and *∆abf2 mhr1-1* mutant cells. (**a**) Mitochondrial nucleoid signals in wild-type, *∆abf2*, *mhr1-1* and *∆abf2 mhr1-1* cells cultivated in YPGly media, or in YPD media to log-phase at 30 °C or 34 °C. Scale bar = 2 µm. (**b**) Numbers of mtDNA-DAPI foci in individual mother cells. The number of individual cells measured for WT, *∆abf2*, *mhr1-1* and *∆abf2 mhr1-1* mother cells cultivated in YPGly at 30 °C was *n* = 14, 8, 7 and 7, respectively. For cells grown in YPD at 30 °C, *n* = 13, 12, 14 and 17, respectively. For cells grown in YPD at 34 °C, *n* = 28, 30, 38 and 27, respectively. Horizontal lines represent the mean number of DAPI foci.

Next, we investigated the relative abundance of mtDNA nucleoids in WT, Δ*abf2* or *mhr1-1* single-mutant, or Δ*abf2 mhr1-1* double-mutant cells selectively grown in YPGly media, or grown in YPD media at 30 °C or 34 °C for more than 20 generations (Fig. 2). In *Δabf2* single-mutants, approximately 27% of CFUs remained ρ^+^ after cultivation in glucose (Fig. 1g), yet all *∆abf2* mother cells we observed displayed mtDNA-derived nucleoid signals after cultivation in YPD at 30 °C (Fig. 2b), suggesting that loss of respiratory function in *∆abf2* cells may be due to mtDNA deletion mutagenesis. In addition, double-mutant *∆abf2 mhr1-1* cells showed the lowest number of mitochondrial nucleoid signals after cultivation in YPD media (Fig. 2a,b), and nucleoid signals were absent from 44.4% of *∆abf2 mhr1-1* mother cells following cultivation at 34 °C, compared to 0% of WT and *mhr1-1* cells and only 10.0% of *∆abf2* mother cells. These results indicate that mtDNA maintenance is defective without Abf2 and fully functional Mhr1. Since we observed that respiratory function and nucleoid abundance generally decrease in *∆abf2 mhr1-1* cells relative to *∆abf2* cells, it is likely Mhr1 plays a role in protecting mtDNA genomic integrity.

### The *mhr1*-null mutation (*Δmhr1*) causes mtDNA fragmentation

**Figure 3:**
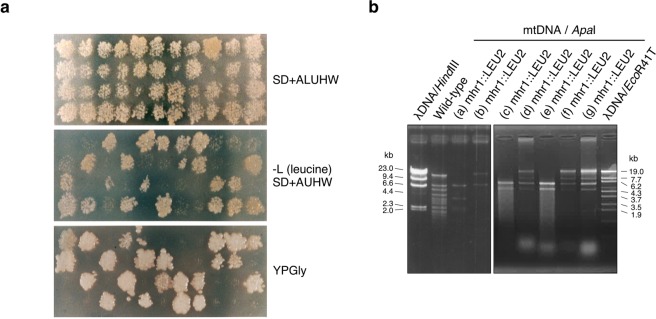
Tetrad analysis of respiratory function and mtDNA deletions in *∆mhr1* cells. (**a**) Respiratory function of ***∆****mhr1* spores was analyzed by replica plating colonies derived from spores (top) onto synthetic media lacking leucine (middle) and YPGly media (bottom). (**b**) *Apa*I digests of purified mtDNA molecules derived from wild-type and ∆*mhr1* spores.

In order to demonstrate that Mhr1 is required to maintain mtDNA integrity, we introduced *mhr1::LEU* DNA fragments into WT/WT diploid cells to disrupt one of the two *MHR1* alleles, thereby creating WT/*Δmhr1* haploinsufficient diploid cells (see: Table 1). We then conducted tetrad analysis to determine whether haploid spores with the nuclear genotype *mhr1::LEU* (*Δmhr1*) retain respiratory function. All leucine prototrophic spores were unable to grow on YPGly plates, confirming that the *Δmhr1* mutation causes complete loss of respiratory function (Fig. 3a). We further cultivated WT cells and seven *Δmhr1* spores (Fig. 3b,a–g) in glucose medium, after selecting cells that still displayed mtDNA signals upon DAPI-staining. *Apa*I-digests of mtDNA from wild-type cells gave rise to many discrete bands (Fig. 3b), while *Apa*I-digests of mtDNA from *Δmhr1* cells gave only a few discrete bands, indicating mtDNA deletions occur in *Δmhr1* cells with some observable amounts of mtDNA remaining.

### MtDNA deletion mutagenesis in ∆*abf2 mhr1-1* cells

**Figure 4:**
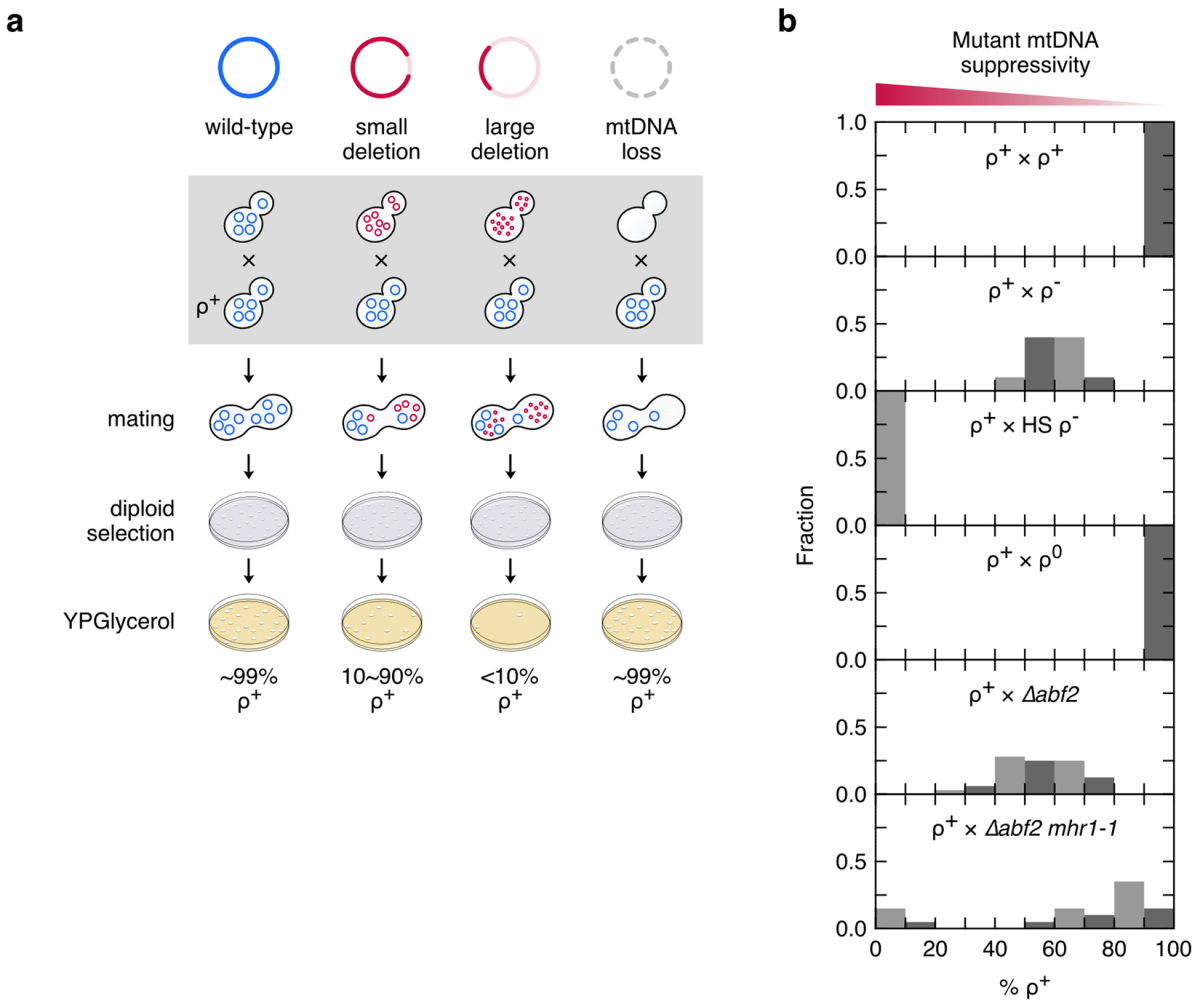
Degree of mtDNA suppressivity in *∆abf2* and *∆abf2 mhr1-1* mutant cells. (**a**) Illustration of the effect of mtDNA deletion size on the suppressive phenotype. (**b**) Frequency distribution of the ρ^+^ phenotype in crosses of ρ^+^, ρ^−^, HS ρ^−^, ρ^0^, *∆abf2* or *∆abf2 mhr1-1* cells (top to bottom, respectively) with ρ^+^ haploid cells (see Table 1). The numbers of independent crosses conducted for each result (shown top to bottom) was, *n* = 5, 10, 5, 5, 32 and 20, respectively.

To determine whether mtDNA deletion mutagenesis or the complete loss of mtDNA occurred in *∆abf2* single- or *∆abf2 mhr1-1* double-mutant cells, we analyzed suppressiveness according to previously established methods^24^. The degree of suppressiveness is determined by: (1) The length of the remnant mtDNA molecule after undergoing a deletion and (2) the preservation of an active *ori* sequence (Fig. 4a). Small mtDNA deletions result in relatively large remnant molecules. Therefore, when crossed with ρ^+^ haploid cells, small mtDNA deletion-bearing cells give rise to diploid populations that are in a range of 10% to 90% ρ^+^. In contrast, when mitochondrial genomes undergo a large deletion event but retain at least one active replication origin, crosses of these haploid cells with ρ^+^ haploid cells of the opposite mating type will give rise to <5% ρ^+^ diploid progeny, a phenotype termed “hypersuppressive”^26^. Finally, crossing haploid cells without mtDNA (ρ^0^) with ρ^+^ haploid cells will result in 100% ρ^+^ diploid progeny, a phenotype termed “non-suppressive” (Fig. 4a).

We crossed a ρ^+^ strain with haploid WT ρ^+^, ρ^−^, HS ρ^−^ or ρ^0^ cells as controls (Fig. 4b, top four panels) and ∆*abf2* or ∆*abf2 mhr1-1* cells, and analyzed the respiratory phenotypes of the resulting diploid progeny. In crosses with the *∆abf2* background, 20~80% of diploid colonies were ρ^+^, with a single distribution centered at around 55% ρ^+^ (Fig. 4b, second panel from bottom). In contrast to this moderately suppressive phenotype, crossing ∆*abf2 mhr1-1* cells yielded a bimodal distribution in which resulting diploid cells either displayed a hypersuppressive, or moderate to non-suppressive phenotype, ranging from 0~20% and 50~100% ρ^+^, respectively (Fig. 4b, bottom panel). These results indicate that large-scale mtDNA deletions or the complete loss of mtDNA occurs in ∆*abf2 mhr1-1* cells, suggesting that the increased production of ρ^−^ progeny (Fig. 1c,g) is due to a deficiency of Mhr1-driven mtDNA recombination.

**Figure 5:**
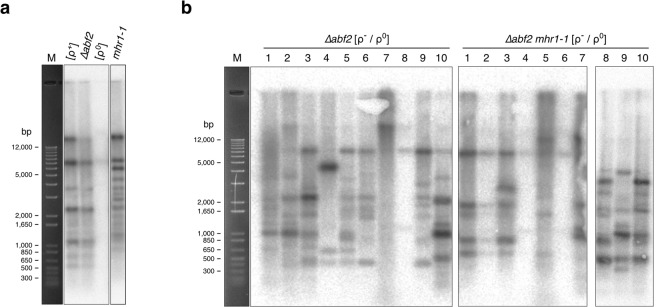
Southern blot analysis of *Apa*I-digested mtDNA from ∆*abf2* or ∆*abf2 mhr1-1* cells. (**a**) MtDNA signals from ρ^+^ WT, *∆abf2*, ρ^0^ and *mhr1-1* cells. (**b**) MtDNA signals from ten ρ^−^/ρ^0^ *∆abf2* colonies and ten ρ^−^/ρ^0^ *∆abf2 mhr1-1* colonies. M: 1-kb plus DNA ladder marker.

Next, we analyzed mtDNA from ρ^−^ *∆abf2* and *∆abf2 mhr1-1* colonies by Southern blot analysis. Compared to ρ^+^ WT and *∆abf2* controls (Fig. 5a), we observed that ρ^−^ *∆abf2* mtDNA generally lacked several mtDNA-specific bands that were present in ρ^+^ mtDNA (Fig. 5b). Similarly, *∆abf2 mhr1-1* double-mutant cells generally lacked many mtDNA-specific bands and some samples lacked mtDNA signals altogether, compared to the mtDNA signals derived from ρ^+^ WT and *mhr1-1* cells (Fig. 5a,b). Also, to our surprise we observed several more small, mtDNA-specific bands in the *mhr1-1* control compared to the ρ^+^ WT and *∆abf2* controls. One explanation is that mtDNA deletions caused by *mhr1-1* mutation result in heteroplasmy, which would be stable since the *mhr1-1* mutation delays mitochondrial allele segregation^20^. Collectively, these results indicate that mtDNA deletions occur in ρ^−^ *∆abf2* cells and that large-scale mtDNA deletion or the complete loss of mtDNA occurs in ρ^−^ or ρ^0^ *∆abf2 mhr1-1* cells.

### Mhr1 overproduction prevents mtDNA deletion mutagenesis

**Figure 6:**
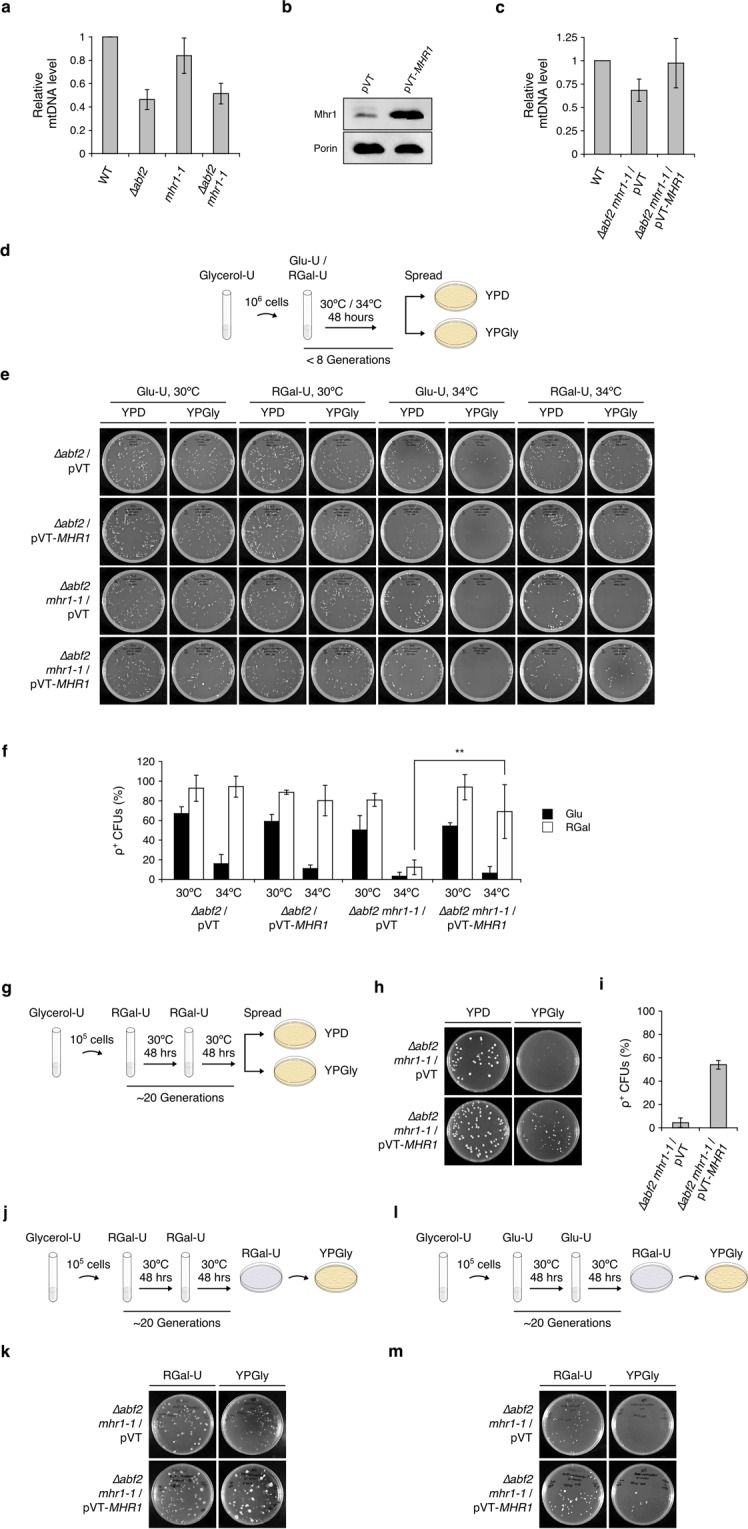
Effects of Mhr1 overproduction on mtDNA content and respiratory function. (**a**) Relative mtDNA level in wild-type, *∆abf2*, *mhr1-1* or *∆abf2 mhr1-1* cells cultivated in YPGly media. (**b**) Immunoblot analysis of Mhr1 protein content in cells containing the pVT or pVT-*MHR1* plasmids. (**c**) Relative mtDNA levels in wild-type or *∆abf2 mhr1-1* cells containing empty or *MHR1*-overexpressing plasmids, after cultivation in Gly-U media. (**d**) Scheme of respiratory function assay. Cells were selectively pre-cultured in Gly-U media, then 10^6^ cells were transferred to Glu-U or RGal-U media and cultivated for <8 generations at 30 °C or 34 °C. Cells were then spread onto YPD and YPGly plates. (**e**) Representative plate images of *∆abf2* and *∆abf2 mhr1-1* CFUs harboring empty or *MHR1*-overexpressing plasmids, following growth in Glu-U or RGal-U media at 30 °C or 34 °C for <8 generations. (**f**) ρ^+^ CFU formation rate based on *n* = 3 independent experiments described in (**d**). (**g**) Scheme of extended respiratory function assay. Cells were selectively grown in Gly-U media, then 10^5^ cells were transferred to RGal-U media and cultivated for two consecutive 48-hour rounds (approximately 20 generations) at 30 °C. Cells were then spread onto YPD and YPGly plates. (**h**) Representative plate images and (**i**) ρ^+^ CFU formation rate based on *n* = 3 independent experiments described in (**g**). (**j**) Scheme of extended respiratory function assay. Cells were selectively grown in Gly-U media, then 10^5^ cells were transferred to RGal-U media and cultivated for two consecutive 48-hour rounds (approximately 20 generations) at 30 °C. Cells were then spread onto RGal-U plates, grown for four to seven days, and then replica-plated onto YPGly plates. (**k**) Representative plate images for *n* = 3 independent experiments as described in (**j**). (**l**) Scheme of extended respiratory function assay. Cells were selectively grown in Gly-U media, then 10^5^ cells were transferred to Glu-U media and cultivated for two consecutive 48-hour rounds (approximately 20 generations) at 30 °C. Cells were then spread onto RGal-U plates, grown for four to seven days, and then replica-plated onto YPGly plates. (**m**) Representative plate images for *n* = 2 independent experiments as described in (**l**). All error bars indicate ± SD.

To verify that Abf2 and Mhr1 are required for mtDNA maintenance, we analyzed mtDNA levels relative to nuclear DNA using quantitative PCR^27^. We observed that the mtDNA level in *∆abf2* cells was less than half (46.3 ± 8.6%) that of WT cells grown in YPGly medium (Fig. 6a). Consistent with our previous results^17^, a large proportion (83.9 ± 15.3%) of mtDNA content was retained in *mhr1-1* cells grown in glycerol medium, while we observed no additive effect on the depletion of mtDNA in *∆abf2 mhr1-1* double-mutant cells (51.4 ± 8.8%), suggesting Abf2 is dispensable for Mhr1-driven mtDNA replication (Fig. 6a).

To investigate the effects of increasing the amount of Mhr1 on mtDNA content and cellular respiratory function, we constitutively overexpressed *MHR1* under the ADH promoter via plasmid in ∆*abf2* single-mutant and ∆*abf2 mhr1-1* double-mutant cells, and confirmed Mhr1 overproduction by immunoblot analysis (Fig. 6b; Supplementary Fig. 1). qPCR analysis revealed that *∆abf2 mhr1-1* double-mutant cells harboring an empty vector had 68.4 ± 11.9% of the mtDNA level of wild-type cells. In contrast, Mhr1 overexpression resulted in an mtDNA level of 97.5 ± 26.5% (Fig. 6c).

To examine whether exogenous *MHR1* expression rescues respiratory function, we compared ∆*abf2* single-mutant and ∆*abf2 mhr1-1* double-mutant cells harboring empty (pVT) or *MHR1*-overexpressing plasmids (pVT-*MHR1*) after 48 hours of growth (equivalent to less than eight generations) in fermentable glucose minus uracil (Glu-U) or raffinose-galactose minus uracil (RGal-U) media at 30 °C or 34 °C (Fig. 6d). We then spread equal amounts of dilute culture on YPD and YPGly plates, as described in Fig. 1. Glucose strongly reduced respiratory growth levels in ∆*abf2*/pVT cells, a result closely matching that in *∆abf2* cells without plasmid DNA (Fig. 1c). ∆*abf2*/pVT cells grown in Glu-U medium produced 67.0 ± 6.9% ρ^+^ colonies at 30 °C and only 16.1 ± 9.4% at 34 °C. In contrast, ∆*abf2*/pVT cells yielded 92.9 ± 13.3% and 94.6 ± 10.8% ρ^+^ colonies when grown in RGal-U at 30 °C or 34 °C, respectively. In agreement with our result from *∆abf2* cells, cellular respiratory function is significantly reduced following cultivation of ∆*abf2*/pVT cells at 34 °C in Glu-U medium, but not in RGal-U. There was a small decrease in respiratory growth upon *MHR1*-overexpression in the ∆*abf2*/pVT-*MHR1* cells in Glu-U or RGal-U media at either 30 °C or 34 °C, indicating that additional Mhr1 is not sufficient to offset a lack of Abf2. Although nucleoid formation defects in the *∆abf2* background cause respiratory defects^28,29^, it is very likely such defects are unable to be prevented by *MHR1*-overexpression. On the other hand, ρ^+^ CFU formation rates were only 50.3 ± 14.8% and 3.3 ± 4.2% in ∆*abf2 mhr1-1*/pVT cells in Glu-U medium at 30 °C, and 34 °C, respectively. Importantly, ∆*abf2 mhr1-1*/pVT cells also displayed highly temperature-sensitive respiratory function after cultivation in RGal-U, giving ρ^+^ CFU formation rates of 81.0 ± 6.6% and 12.4 ± 7.4% at 30 °C and 34 °C, respectively. Addition of *MHR1* in ∆*abf2 mhr1-1*/pVT-*MHR1* cells significantly rescued the respiratory function of these cells in RGal-U at 34 °C, to a ρ^+^ CFU formation rate of 69.1 ± 27.5%. On the other hand, ∆*abf2 mhr1-1*/pVT-*MHR1* cells cultivated in Glu-U showed no rescue effect, with ρ^+^ CFU formation rates of 54.5 ± 3.2% and 6.3 ± 6.8% at 30 °C and 34 °C, respectively (Fig. 6e,f).

**Figure 7:**
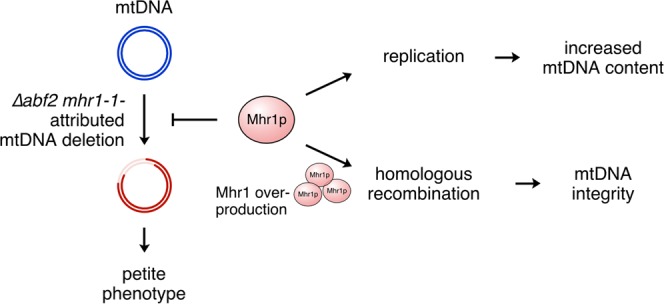
Model for the prevention of mtDNA deletion mutagenesis by Mhr1-driven recombination and mtDNA replication. MtDNA deletions threatening cellular respiratory function arising from genetic backgrounds such as *∆abf2 mhr1-1* can be prevented by increased homologous recombination via overproduction of Mhr1 recombinase.

To further advance the notion that Mhr1 can protect mtDNA integrity, we examined the effect of an extended cultivation time in fermentable media of approximately 20 generations. Simultaneous spreading of ∆*abf2 mhr1-1*/pVT and ∆*abf2 mhr1-1*/pVT-*MHR1* cells onto YPD and YPGly plates (Fig. 6g) yielded 4.5 ± 4.1% and 54.0 ± 3.6% ρ^+^ CFUs, respectively (Fig. 6h,i), reinforcing the notion of a rescue effect for additional Mhr1 in RGal-U media. Similarly, replica-plating ∆*abf2 mhr1-1*/pVT and ∆*abf2 mhr1-1*/pVT-*MHR1* colonies from RGal-U to YPGly plates showed a clear increase in the proportion of ρ^+^ CFUs upon *MHR1* overexpression (Fig. 6j,k), while only a small proportion of CFUs remained ρ^+^ after cultivation in Glu-U medium (Fig. 6l,m). These results indicate that glucose impairs Mhr1-mediated action, which protects against mtDNA deletions. In summary, Mhr1 functions to prevent loss of respiratory function due to mtDNA deletion mutagenesis, although deletions are not completely prevented by overproduced Mhr1 (Fig. 7).

## Discussion

In this study, we found that increasing Mhr1 protein level prevents loss of respiratory function in cells lacking Abf2 and functional Mhr1, which display an mtDNA-instability phenotype similar to several other nuclear mutations in yeast. Our results provide further support for the notion that Mhr1 has a pivotal role in the maintenance of mitochondrial genomic integrity^30^, and that DSB-induced mtDNA replication by Mhr1 is the predominant form of mtDNA replication in ρ^+^ yeast cells^23^. We previously reported that Mhr1-dependent mtDNA replication and homologous recombination are crucial for repair of mtDNA DSBs^22^. Collectively, our results here suggest that mitochondrial homologous DNA recombination may have utility in preventing the spontaneous generation of mtDNA deletions in a variety of circumstances (Fig. 7).

The requirement for Abf2 and Mhr1 in mtDNA stability raises the question of how increasing the amount of Mhr1 alone prevents generation of deleted mtDNA and helps to sustain cellular respiratory function. The answer likely comes from one of the mechanisms of mtDNA deletion formation proposed by Krishnan *et al*., in which mtDNA deletions occur during repair of DSB-induced mtDNA damage, rather than during replication^31^. We inferred that a lack of Abf2 might weaken DSB repair by homologous recombination and increase the accumulation of deleted mtDNA molecules. Since DSB repair can be accomplished by homologous DNA recombination^32–34^, an increased amount of Mhr1 may enhance the number of homologous DNA recombination events to repair DSBs^22^. This interpretation implies that mtDNA deletions are prevented, mtDNA integrity is maintained, and a large proportion of cells with the ∆*abf2* mutation sustain respiratory function upon overexpression of *MHR1* (Fig. 6). On the other hand, since mtDNA deletion mutagenesis still occurs upon Mhr1 overproduction in *∆abf2* cells, the stabilization of mtDNA recombination intermediates by Abf2^14^ is important. In addition, we found that a fraction of the population of *∆abf2 mhr1-1* cells contains HS ρ^−^ mtDNA molecules (less than 20%; Fig. 4b), however this proportion is likely large enough to reduce the beneficial effect of Mhr1 overexpression on preventing deleterious mtDNA mutations. Due to the presence of HS ρ^−^ mtDNA mutant molecules, Mhr1 overexpression likely increases the amounts of both wild type and HS ρ^−^ mtDNA. Thus, it is very unlikely that the observed rescue of *∆abf2 mhr1-1* cells upon *MHR1* overexpression (Fig. 6) is due only to increased replication and selection for wild-type mtDNA over deleterious mtDNA mutations. These results therefore indicate that increased amounts of Mhr1 can prevent mitochondrial genomic instability.

Mhr1 promotes homologous DNA pairing^18,19^, while Abf2 packages mtDNA and is the main component of the nucleoid^7,35^. Both of these molecular functions resemble that of RecA in *Escherichia coli*. RecA forms a nucleoprotein complex that both ensures the formation of heteroduplex DNA and protects DNA from nuclease degradation^36^. Since these roles are performed by discrete proteins in *S*. *cerevisiae* mitochondria, whether mtDNA-Mhr1 nucleoprotein formation occurs prior to packaging of concatemeric mtDNA molecules by Abf2 or by a contrary process, remains for further investigation.

TFAM, the mammalian ortholog of Abf2, contains an additional C-terminal domain that has been predicted to be essential for transcription^13^. TFAM can complement Abf2 in yeast by rescuing the loss-of-mtDNA phenotype of yeast *∆abf2* cells^37^. In contrast to Abf2/TFAM, the existence of a metazoan ortholog of Mhr1 remains an open issue^38^. To date, there have been several lines of evidence to indicate that human mtDNA recombination may occur^39–41^. For example, we demonstrated that ROS stimulate mitochondrial allele segregation from heteroplasmy towards homoplasmy in human fibroblasts^42^, a result consistent with the stimulatory effect of ROS and mechanism of recombination-driven mtDNA replication in yeast mitochondria^21,43^. Consistent with the results reported here, a method to stimulate recombination function in human mitochondria may similarly prevent human mtDNA instability and present numerous other health benefits.

## Materials and Methods

### Yeast strains and media

Yeast strains used in this study are listed in Table 1. General genetic techniques used in this study are described in^44^. Yeast transformation was carried out using the lithium-acetate method^45^. The assay for hypersuppressiveness was carried out according to a procedure previously described^24,46^. Overexpression of *MHR1* was achieved using a yeast multiple-copy plasmid with a constitutive promoter (pVT100U)^47^. The ORF (open reading frame) of *MHR1* was amplified by PCR with addition of *Sac1* and *Xba1* restriction sites at the 5′ and 3′ ends respectively, and inserted into pVT100U to generate pVT100U-*MHR1*. Immunoblot analysis for Mhr1 detection was performed using rabbit antiserum against Mhr1, according a previously established procedure^22^.

Media were prepared as previously described^17,18,24^. Selective pre-cultivation of cells for respiratory function assays was conducted either in rich glycerol (YPGly), synthetic glycerol (Gly) or synthetic glycerol minus uracil (Gly-U) media. Fermentable media, used to promote loss of respiratory function, was rich glucose (YPD), synthetic glucose (Glu), synthetic glucose minus uracil (Glu-U), synthetic raffinose-galactose (RGal), or synthetic raffinose-galactose minus uracil (RGal-U). Glycerol, glucose, raffinose and galactose concentrations used were 3% v/v, 2%, 2% and 2%, respectively. Selection for diploid cells following crossing with *∆abf2* or *∆abf2 mhr1-1* cells was carried out by cultivating cells in liquid synthetic minimal (SD) or synthetic minimal plus tryptophan (SD + W) medium containing 2% glucose at 30 °C overnight and then spreading cells on agar plates containing the same nutrients. For control crosses, diploids were selected by spreading mated cells directly onto synthetic minimal media agar plates containing leucine plus uracil (SD + LU) or tryptophan (SD + W) and 2% glucose.

### Mitochondrial nucleoid analysis

WT, *mhr1-1*, *∆abf2* and *∆abf2 mhr1-1* cells were cultivated in YPGly media to early log-phase at 30 °C and stained with DAPI. Cells were subsequently transferred to YPD media and cultivated for two consecutive rounds at 30 °C or 34 °C, stained with DAPI and observed again. DAPI staining was performed by transferring aliquots to fresh media containing 1 µg/ml DAPI and incubating for 15 min. Cells were then washed and resuspended in fresh media and 1% low-melting point agarose at a 1:1 ratio. Cells were then mounted on glass slides and analyzed with a Deltavision fluorescence microscopy system (Applied Precision, Inc.) equipped with an Olympus IX71 microscope. DAPI foci in mother cells were counted using ImageJ software.

### Tetrad analysis

We used an Olympus micromanipulation system for tetrad dissection to separate the four ascus-encapsulated spores derived from diploid WT/*Δmhr1* cells. Spores were separated and placed on synthetic defined (SD) complete plates supplemented with adenine (A), leucine (L), uracil (U), histidine (H) and tryptophan (W), and cultivated at 30 °C for 7 days. Colonies were then replica-plated onto SD complete plates lacking L and YPGly plates.

### Purification of yeast mtDNA and analysis by restriction enzyme digestion

MtDNA was purified from *Δmhr1* spore-derived yeast cells using cesium chloride density-gradient centrifugation^48^. The purified mtDNA was digested with *Apa*I and run on a 1% agarose gel alongside DNA size markers. The DNA fragments were photographed under ultraviolet irradiation after staining the gel with 0.5 μg/ml ethidium bromide.

### Southern blot analysis

∆*abf2* and ∆*abf2 mhr1-1* cells were cultivated in YPGly media, then transferred to RGal complete media and cultivated at 30 °C for two days. Total cellular DNA was then prepared and digested by *Apa*I. Approximately 15 µg of total DNA was separated by electrophoresis on a 1.0% agarose gel, run at 24 °C for 80 h at 5 V/cm, and transferred to a nylon membrane (Amersham Hybond N Plus; GE Healthcare). Signals for mtDNA were detected using ^32^P-labeled full-length mtDNA from budding yeast as a probe. Signals were analyzed using a Typhoon FLA 7000 biomolecular imager (GE Healthcare).

### Analysis of mtDNA level by quantitative real-time PCR

Primers used for real-time PCR were as follows: COX3-Forward, 5′-TCCATTCAGCTATGAGTCCTGATG-3′; COX3-Reverse, 5′-AATTCGGTAGGTTGTACAGCTTCAA-3′; NUC1-Forward, 5′-TTTAGGTCGGGCTATGATCGA-3′; NUC1-Reverse, 5′-TCCATGGCCTGTTGAGAAAAT-3′. The 20 µl reaction volumes contained 10 µl SYBR Premix Ex Taq^TM^ II (Takara), 0.4 µM of each primer, and 100 ng of template genomic DNA. A LightCycler 480 (Roche) was used for real-time PCR analysis^27^.

## Supplementary information

Supplementary Figures
