# Supplementary material for: Prevention of mitochondrial genomic instability in yeast by the mitochondrial recombinase Mhr1
Source: Sci Rep. 2019 Apr 1;9:5433. doi: 10.1038/s41598-019-41699-9 (PMC6443803; doi:10.1038/s41598-019-41699-9)
Supplement: Supplementary file 1 — Supplementary Figures [file 41598_2019_41699_MOESM1_ESM.pdf]

Submitted to Scientific Reports (SREP-18-06564A)

Supplementary materials for:

Prevention of mitochondrial genomic instability in yeast by the mitochondrial recombinase Mhr1

Feng Ling[*], Elliot Bradshaw[*,†], Minoru Yoshida[*,†]

[*]Chemical Genomics Research Group, RIKEN Center for Sustainable Resource Science, Hirosawa 2-1, Wako, Saitama 351-0198, Japan;

[†]Graduate School of Science and Engineering, Saitama University, Saitama 338-8570, Japan

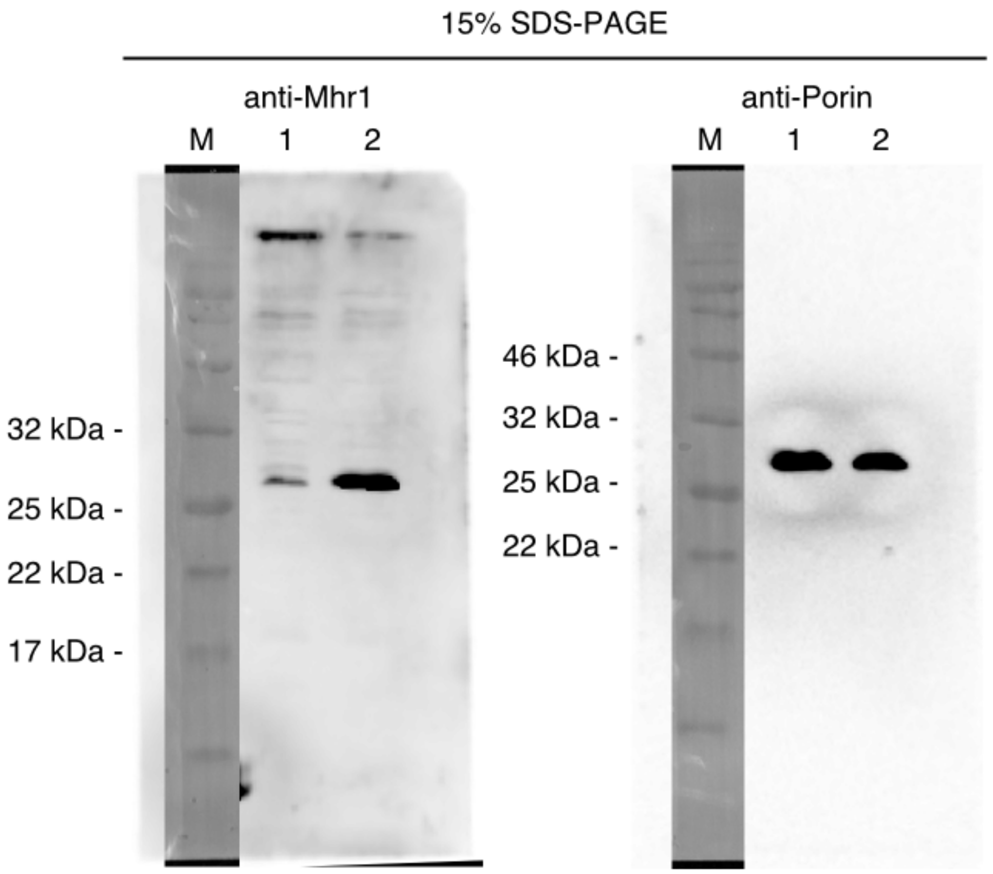

Supplementary Figure 1. Full-length immunoblot images for analysis of Mhr1 overproduction. Cells were cultivated in synthetic defined (SD) Gly-URA media to early log-phase. Cell-free extracts were then prepared from Δ*abf2 mhr1-1* cells containing an empty plasmid (1; pVT100U-Empty) or a plasmid overproducing Mhr1 (2; pVT100U-*MHR1*) using the LiAc / NaOH method[2]. Denatured proteins were subjected to sodium dodecyl sulfate-polyacrylamide gel electrophoresis. Immunoblot analysis was performed using anti-Mhr1 serum to probe Mhr1 and anti-Porin to represent mitochondrial protein amounts as described[1]. 5 µl blue prestained protein standard (NEB #P7706) was used as a molecular weight marker (M).

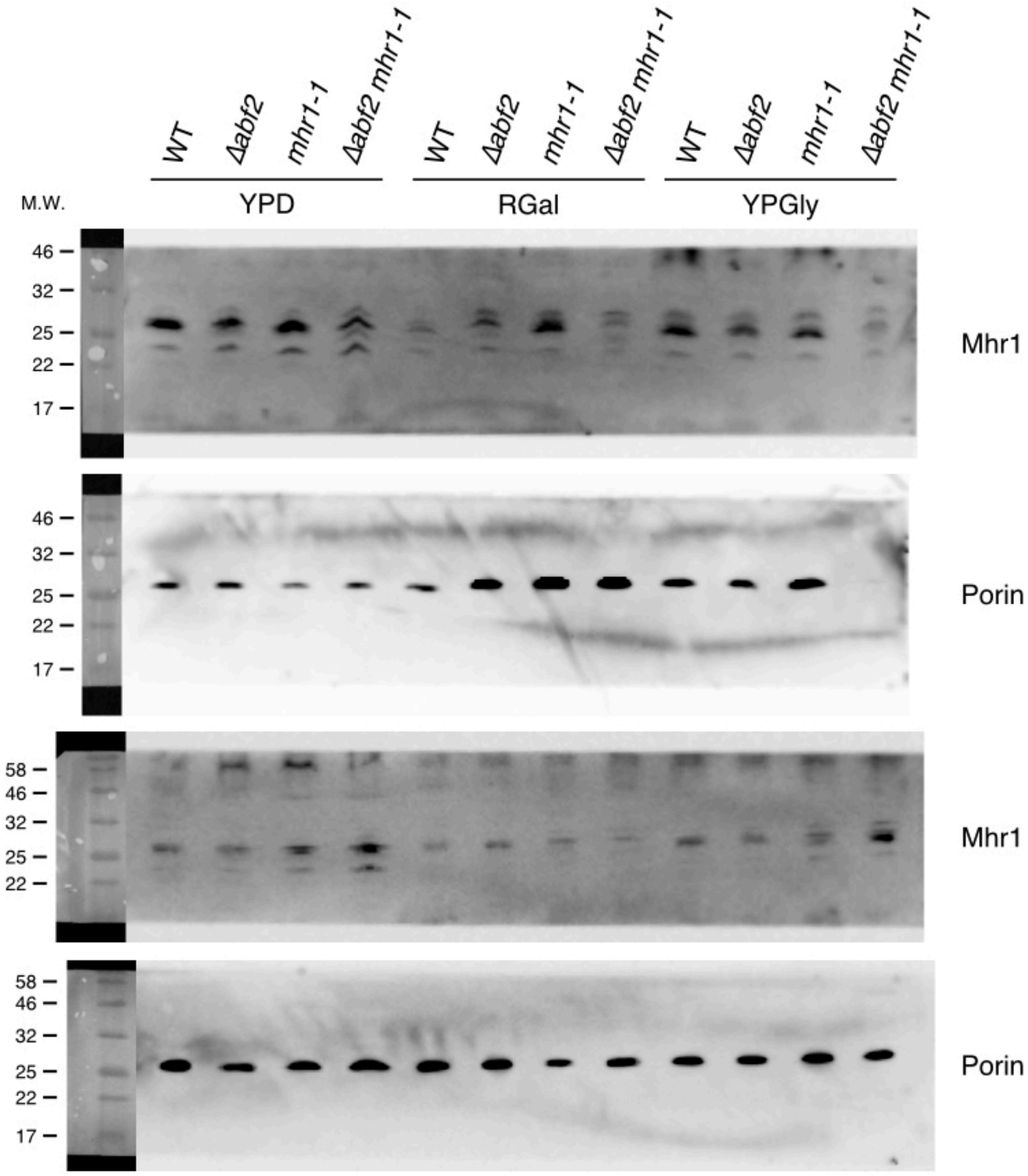

Supplementary Figure 2. Immunoblot analysis of Mhr1 protein levels in different media. Cell-free extracts were prepared from cells cultivated in YPD, RGal and YPGlycerol media using the LiAc / NaOH method[2]. Results shown are from two independent experiments. Molecular weights (kDa) are indicated on the left.

References:

1. Ling, F. & Shibata, T. Recombination-dependent mtDNA partitioning: in vivo role of Mhr1p to promote pairing of homologous DNA. *EMBO J* **21**, 4730-4740

(2002).

2. Zhang, T., Lei, J., Yang, H., Xu, K., Wang, R. *et al.* (2011). An improved method for whole protein extraction from yeast *Saccharomyces cerevisiae*. *Yeast* 28: 795-798.
